# Differential transcriptional modulation of duplicated fatty acid-binding protein genes by dietary fatty acids in zebrafish (*Danio rerio*): evidence for subfunctionalization or neofunctionalization of duplicated genes

**DOI:** 10.1186/1471-2148-9-219

**Published:** 2009-09-02

**Authors:** Santhosh Karanth, Santosh P Lall, Eileen M Denovan-Wright, Jonathan M Wright

**Affiliations:** 1 Department of Biology, Dalhousie University, Halifax, Nova Scotia, B3H 4J1, Canada; 2 National Research Council of Canada, Institute of Marine Biosciences, Halifax, Nova Scotia, B3H 3Z1, Canada; 3 Department of Pharmacology, Dalhousie University, Halifax, Nova Scotia, B3H 1X5, Canada

## Abstract

**Background:**

In the Duplication-Degeneration-Complementation (DDC) model, subfunctionalization and neofunctionalization have been proposed as important processes driving the retention of duplicated genes in the genome. These processes are thought to occur by gain or loss of regulatory elements in the promoters of duplicated genes. We tested the DDC model by determining the transcriptional induction of fatty acid-binding proteins (Fabps) genes by dietary fatty acids (FAs) in zebrafish. We chose zebrafish for this study for two reasons: extensive bioinformatics resources are available for zebrafish at zfin.org and zebrafish contains many duplicated genes owing to a whole genome duplication event that occurred early in the ray-finned fish lineage approximately 230-400 million years ago. Adult zebrafish were fed diets containing either fish oil (12% lipid, rich in highly unsaturated fatty acid), sunflower oil (12% lipid, rich in linoleic acid), linseed oil (12% lipid, rich in linolenic acid), or low fat (4% lipid, low fat diet) for 10 weeks. FA profiles and the steady-state levels of *fabp *mRNA and heterogeneous nuclear RNA in intestine, liver, muscle and brain of zebrafish were determined.

**Result:**

FA profiles assayed by gas chromatography differed in the intestine, brain, muscle and liver depending on diet. The steady-state level of mRNA for three sets of duplicated genes, *fabp1a/fabp1b.1/fabp1b.2*, *fabp7a/fabp7b*, and *fabp11a*/*fabp11b*, was determined by reverse transcription, quantitative polymerase chain reaction (RT-qPCR). In brain, the steady-state level of *fabp7b *mRNAs was induced in fish fed the linoleic acid-rich diet; in intestine, the transcript level of *fabp1b.1 *and *fabp7b *were elevated in fish fed the linolenic acid-rich diet; in liver, the level of *fabp7a *mRNAs was elevated in fish fed the low fat diet; and in muscle, the level of *fabp7a *and *fabp11a *mRNAs were elevated in fish fed the linolenic acid-rich or the low fat diets. In all cases, induction of the steady-state level of *fabp *mRNAs by dietary FAs correlated with induced levels of hnRNA for a given *fabp *gene. As such, up-regulation of the steady-state level of *fabp *mRNAs by FAs occurred at the level of initiation of transcription. None of the sister duplicates of these *fabp *genes exhibited an increase in their steady-state transcript levels in a specific tissue following feeding zebrafish any of the four experimental diets.

**Conclusion:**

Differential induction of only one of the sister pair of duplicated *fabp *genes by FAs provides evidence to support the DDC model for retention of duplicated genes in the zebrafish genome by either subfunctionalization or neofunctionalization.

## Background

In his seminal book, 'Evolution by Gene Duplication', Susumu Ohno [1] argued that one of the major mechanisms that facilitate the increasing complexity in the evolution of life is duplication of genes and whole genomes. Models to explain retention of duplicated genes in eukaryotic genomes have undergone a development of thought since Ohno proposed his model almost 40 years ago. Ohno [1] argued that most duplicated genes are lost from the genome owing to nonfunctionalization, a claim which has been validated by empirical evidence from Lynch and Conery [2]. Nonfunctionalization is a process where deleterious mutations accumulate in the coding region of a gene giving rise to either a dysfunctional protein or no protein product. Duplicated genes might, however, be retained in the genome owing to mutations in the coding region that led to a novel function for the protein product of a gene, a process Ohno termed 'neofunctionalization'. This model by Ohno for the preservation of duplicated genes came to be known as the 'classical model'. Data primarily derived from genome sequencing projects over the past decade suggest that a much higher proportion of gene duplicates is preserved in the eukaryotic genome than predicted by Ohno's "classical model". To explain this apparent "conundrum", Force *et al*. [3] proposed the Duplication-Degeneration-Complementation (DDC) model in which subfunctionalization serves as an alternative mechanism, but not to the exclusion of neofunctionalization, for the preservation of duplicated genes. According to the DDC model, duplicated genes are retained in the genome either by subfunctionalization, where the functions of the ancestral gene are sub-divided between sister duplicate genes, or by neofunctionalization, where one of the duplicates acquires a new function. In this model, both processes occur by either loss or gain of *cis*-acting regulatory elements in the promoters of the duplicated genes. As with the "classical model" of Ohno [1], the DDC model proposes that most duplicated genes are lost from the genome (*i.e*., nonfunctionalization) owing to an accumulation of deleterious mutations in coding or control regions leading to functional decay.

We chose to test the DDC model of Force *et al*. [3] that subfunctionalization or neofunctionalization results in the retention of duplicated genes in the genome by investigating the expression of duplicated copies of fatty acid-binding protein (Fabp) genes, members of the multigene family of intracellular lipid-binding protein (iLBP) genes, in zebrafish for two reasons. First, bioinformatic resources for zebrafish are readily available, including linkage maps, extensive expressed sequence tags (EST)  and an almost complete genome sequence database . Second, and most importantly, owing to a whole genome duplication (WGD) event, an event that occurred early in the ray-finned fish radiation about 230-400 million years ago [4-7], we predicted that many members of the iLBP multigene family might exist as duplicated copies. This prediction has proved to be correct (see below).

The iLBPs are encoded by a highly conserved multigene family, which consists of the fatty acid- (FABP), cellular retinol- (CRBP) and cellular retinoic acid-binding protein (CRABP) genes [reviewed in [8-12]]. Currently, 17 paralogous iLBP genes have been identified in animals, but no member of this multigene family has been identified in plants and fungi. Schaap *et al*. [13] have suggested, therefore, that the first iLBP gene emerged after the divergence of animals from plants ~930-1000 million years ago. This ancestral iLBP gene presumably underwent a series of duplications followed by sequence divergence, giving rise to the diversity of the extant iLBP multigene family. This multigene family has been further augmented in ray-finned fishes by the WGD event mentioned above [4-7].

Originally, iLBP genes and their proteins were named according to the initial tissue of isolation, *e.g*., liver-type FABP (L-FABP), brain-type FABP (B-FABP), *etc*. Owing to some confusion with this earlier nomenclature, here we use the nomenclature proposed by Hertzel and Bernlohr [14] in which numerals distinguish the different FABP proteins and their genes, (*e.g*., FABP1 (liver-type), FABP7 (brain-type)).

FABP1, the first FABP isolated, was described almost 40 years ago [15]. Although extensive studies in mammals have focused on the tissue distribution and binding activities of FABPs, the regulation and evolution of their genes, and mice FABP gene knock-outs [8,10-12], the precise physiological roles of FABPs remain unclear. However, accumulated data have provided evidence that FABPs play an important role in uptake, sequestering and transport of fatty acids and interaction with other transport and enzyme systems. Indirect evidence suggests other putative physiological functions for FABPs, such as: (i) transport of fatty acids to the nucleus to regulate gene transcription via activation of the nuclear receptors, the peroxisome proliferator-activated receptors (PPAR) [see *e.g*., [12,16]]; (ii) essential functions in early development, especially neural growth and differentiation [see [17,18] and references therein]; and (iii) a role in human diseases [10,12].

Although the coding sequence and structure of the FABP genes has been well conserved over millions of years, each FABP gene exhibits a distinct, yet sometimes overlapping pattern of tissue-specific expression with other FABP genes. If all FABP genes in this multigene family arose from a single ancestral gene as proposed [13], the regulatory elements in the promoters must have evolved different functions in the subsequent duplicated genes giving rise to the complexity of the spatio-temporal expression of this multigene family. Regulatory elements in the promoters of some mammalian FABP genes and one insect have been identified [8,19]. As such, *cis*-acting elements that determine the spatio-temporal expression of these genes, with a few exceptions, are not well defined. From sequence data, it appears that mammalian iLBP gene-promoters consist of a modular structure similar to many eukaryotic promoters, comprised commonly of a TATA box with proximal and distal regulatory elements [20]. Currently, our understanding of the regulatory elements that control the expression of the FABP genes is modest and limited primarily to mammals [but see [21-23]]. Based on the zebrafish *fabp10 *sequence [24], Her *et al*., [22] cloned its promoter and by functional analysis, identified a 435 bp regulatory element that is sufficient to modulate the liver regional expression in transgenic zebrafish. How this *cis-*acting element functions is not known. In addition to functional promoter studies, fatty acids and peroxisome proliferators have been shown to induce the transcription of some FABP genes in mammals [25,26] via activation of PPAR, or other unknown transcription factors, that bind to a fatty acid (FA) response element (FARE) [23].

To date, we have characterized 11 zebrafish FABP genes with respect to their cDNA sequence, gene structure, chromosome location, conserved gene synteny with their mammalian orthologs, and their spatio-temporal patterns of expression in embryos, larvae and adults [17,18,24,27-34]. Based on phylogenetic analyses and conserved gene synteny with their single-copy, mammalian orthologs, eight (four pairs) out of 11 of the extant members of the zebrafish FABP genes arose as a result of the ray-finned fish-specific WGD event [[18,32,33] and unpublished data]. One pair of genes, *fabp1b.1 *and *fabp1b.2*, are tandemly-arrayed on chromosome 8 separated by ~4 kb of DNA [unpublished data]. This duplication, which was subsequent to the WGD but is not yet dated, is presumably the result of unequal crossing-over between homologous chromosomes during meiosis. The number of duplicated FABP genes (63%) retained in the zebrafish genome owing to the WGD event in the ray-finned fishes lineage is remarkable as Postlethwaith *et al*. [4] estimate that only 20% of all the duplicated genes following WGD have been retained in the zebrafish genome. Other estimates for retention of duplicated genes in the zebrafish genome from the WGD are 14-30% [6]. Three zebrafish FABP genes, *fabp2, fabp3 *and *fabp6 *exist as single copies (a duplicate of *fabp10 *has recently been identified by us [unpublished data]). Following the WGD event, the sister duplicates of these genes have presumably been lost by accumulation of mutations leading to functional decay.

In mammals, FAs up-regulate the expression of some FABP genes as evidenced by an increase in mRNA and protein levels [25,26]. We hypothesized that (i) zebrafish *fabp *genes might be up-regulated by dietary FAs, and (ii) sister duplicated *fabp *genes might be differentially modulated by dietary FAs. We show by assaying steady-state mRNA and heterogeneous nuclear RNA (hnRNA) levels for three sets of duplicated *fapb *genes, *fabp1a/fabp1b.1/fabp1b.2, fabp7a/fabp7b *and *fabp11a/fabp11b*, that dietary FAs modulate the transcriptional initiation of only one of the duplicated *fabp *genes in specific tissues of zebrafish. This result provides compelling evidence that these duplicated *fabp *genes were retained in the genome by either subfunctionalization or neofunctionalization owing to the divergence of *cis*-acting regulatory elements in the *fabp *gene promoters.

## Results

### The steady-state level of *fabp *mRNAs does not differ between sexes of zebrafish

Several authors have argued that lipid metabolism is influenced by the sex of an organism. For example, long-chain fatty acids are cleared from plasma by the liver more rapidly in female rats than male rats [35]. Bass *et al*. [36] reported that female rats have a higher intracellular concentration of FABP3 than male rats. We, therefore, assayed by reverse transcription, quantitative polymerase chain reaction (RT- qPCR) the steady-state level of mRNA encoded by each *fabp *gene in intestine, liver, muscle and brain of male and female zebrafish reared on Tetramin^® ^(Tetrawerke, Melle, Germany) flake diet. No difference in the steady-state level of *fabp *mRNAs in a specific tissue (*i.e*., intestine, liver, muscle or brain) was observed between male and female zebrafish (data not shown).

### Effect of diet on the FA composition in different tissues of zebrafish

**Figure 1:**
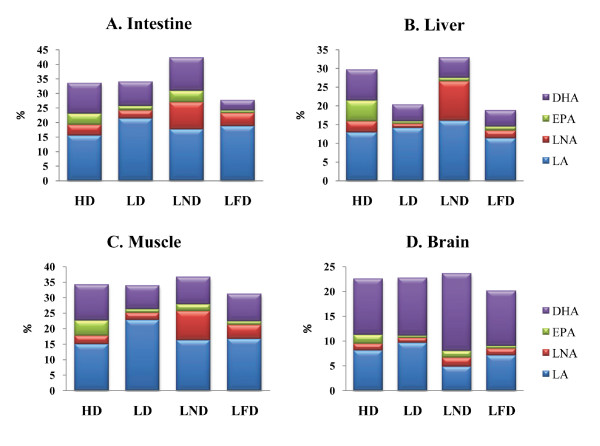
**Fatty acid composition of the four major fatty acids, linoleic acid (18:2 n-6, LA), linolenic acid (18:3 n-3, LNA), eicosapentaenoic acid (20:5 n-3, EPA), and docosahexaenoic acid (22:6 n-3, DHA) in intestine (A), liver (B), muscle (C), and brain (D) of zebrafish fed either the highly unsaturated FA-rich diet (HD), the linoleic acid-rich diet (LD), the linolenic acid-rich diet (LND), or the low fat diet (LFD) for 10 weeks**. Data expressed as area percentage of fatty acid methyl esters (n = 3). Comprehensive FA profiles for intestine (S1), liver (S2), muscle (S3), and brain (S4) of fish fed the four diets are provided as supplementary material (Additional file 1).

Zebrafish readily ate all four diets and no differences were observed in their feed intake during the 10 week feeding period. Composition and abbreviated names for the four diets are shown in Table 1. The weight of fish increased from 0.26 ± 0.02 g at the beginning of the feeding trial (~22 weeks of age) to 0.45 ± 0.01 g at the end of the feeding trial (~32 weeks of age) for all diets. At the end of 10 weeks, each diet resulted in a different FA profile for specific tissues. Fig 1 shows the composition of four major FAs in intestine, liver, muscle and brain. For detailed composition of FAs in each tissue see additional file 1: Tables S1-S4. Intestine, liver, muscle and brain of fish fed HD and LND had higher proportions of n-3 fatty acids, due largely to elevated levels of linolenic acid (18:3 n-3), eicosapentaenoic acid (20:5 n-3), and docosahexaenoic acid (22:6 n-3), and reduced proportions of n-6 fatty acids. In contrast, intestine, muscle, and brain of fish fed LD and LFD exhibit higher percentages of n-6 fatty acids, linoleic acid (18:2 n-6) and arachidonic acid (20:4 n-6), and reduced proportions of n-3 fatty acids. The percentages of other fatty acids varied among tissues within each experimental group and also among the experimental groups (see Additional file 1: Tables S1-S4).

**Table 1:** Composition of experimental diets (g/100 g of dry diet)

**Ingredient**	HD*	LD*	LND*	LFD*
Vitamin free Casein^1^	33	33	33	33
Wheat Gluten^2^	10	10	10	10
Gelatin^1^	4	4	4	4
Flax oil^3^	2	2	8	2
Coconut oil^3^	2	2	2	-
Omega Mix^4^	6	-	-	-
Sunflower oil^3^	2	8	2	2
Corn starch (pre-gelatinized)^5^	29.1	29.1	29.1	37.1
Celufil^1^	8	8	8	8
Vitamin Mix^6^	1.3	1.3	1.3	1.3
Mineral Mix^7^	1	1	1	1
Betaine^8^	1.5	1.5	1.5	1.5
DL-Methionine^1^	0.2	0.2	0.2	0.2
Total	100	100	100	100

* HD - highly unsaturated fatty acids rich diet; LD - linoleic acid rich diet; LND - linolenic acid rich diet; LFD - low fat diet; BHA-butyl hydroxy anisole.

^1^US Biochemical, Cleveland, OH, USA.

^2^Dover Mills Ltd. (Halifax, NS, Canada).

^3^Obtained from the local market.

^4^Jamieson omega-3 select capsules.

^5^National Starch and Chemical Co. (Bridgewater, NJ, USA).

^6^Vitamin added to supply the following (per kg diet): vitamin A, 8000 IU; vitamin D_3_, 4000 IU; vitamin E, 300 IU; vitamin K_3_, 40 mg; thiamine HCl, 50 mg; riboflavin, 70 mg; d-Ca pantothenate, 200 mg; biotin, 1.5 mg; folic acid, 20 mg; vitamin B_12_, 0.15 mg; niacin, 300 mg; pyridoxine HCl, 20 mg; ascorbic acid, 300 mg; inositol, 400 mg; choline chloride, 2000 mg; butylated hydroxy toluene, 15 mg; butylated hydroxy anisole, 15 mg.

^7^Mineral added to supply the following (per kg diet): manganous sulphate (32.5% Mn), 40 mg; ferrous sulphate (20.1% Fe), 30 mg; copper sulphate (25.4% Cu), 5 mg; zinc sulphate (22.7% Zn), 75 mg; sodium selenite (45.6% Se), 1 mg; cobalt chloride (24.8% Co), 2.5 mg; sodium fluoride (42.5% F), 4 mg.

^8^Betaine anhydrous (96% feed grade), Finnfeeds, Finland.

### Effect of diet on the steady-state level of *fabp1a/fabp1b.1/fabp1b.2 *mRNAs in different tissues

**Figure 2:**
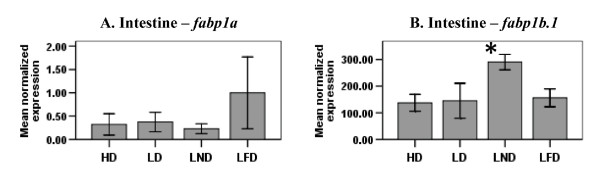
**The steady-state level of *fabp1a *and *fabp1b.1 *mRNA in the intestine of zebrafish fed diets differing in FA content**. The level of *fabp1a *(A) and *fabp1b.1 *(B) mRNA in the intestine of fish fed either diet HD, LD, LND or LFD was determined by RT-qPCR using gene-specific primers. The steady-state level of *fabp *transcripts was normalized to the steady-state level of *ef1α *transcripts in the same samples. Data are presented as the mean ratio ± S.E.M. (x 10^-3^). Significant differences in the relative steady-state level of *fabp *mRNA between fish (n = 6) fed different diets are indicated by an asterisk.

No modulation in the steady-state level of *fabp1a *mRNAs was observed in the intestine (Fig. 2A) regardless of diet fed to the fish. In the intestine of fish fed LND, the steady-state level of *fabp1b.1 *mRNAs was higher than those in the intestine of fish fed the other three diets (Fig. 2B). The level of *fabp1a *and *fabp1b.1 *mRNAs was below that quantifiable by RT-qPCR in liver, muscle and brain of zebrafish. *fabp1b.2 *mRNA was detectable in intestine and brain of fish fed the four diets, but the levels in liver and muscle were below the quantifiable range in RT-qPCR assays (data not shown). Furthermore, dietary FAs did not change the steady-state level of *fabp1b.2 *transcripts in the intestine and brain.

### Effect of diet on the steady-state level of *fabp7a* and *fabp7b *mRNAs in different tissues

**Figure 3:**
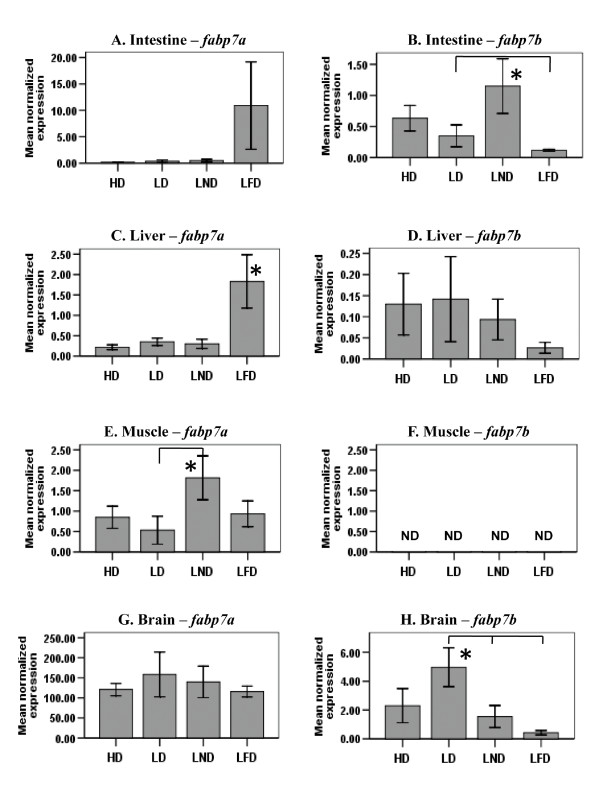
**The steady-state level of *fabp7a *and *fabp7b *mRNA in intestine (A, B), liver (C, D), muscle (E, F), and brain (G, H) of zebrafish fed diets differing in FA content**. The level of *fabp7a *and *fabp7b *mRNA in intestine, liver, muscle, and brain of fish fed either diet HD, LD, LND, or LFD was determined by RT-qPCR using gene-specific primers. The steady-state level of *fabp *transcripts was normalized to the steady-state level of *ef1α *transcripts in the same samples. Data are presented as the mean ratio ± S.E.M. (x 10^-3^). Significant differences in the relative steady-state level of *fabp *mRNA between fish (n = 6) fed different diets are indicated by an asterisk. *fabp7b *mRNA was not detected (ND) in muscle (F).

The steady-state level of *fabp7a *mRNAs was not modulated in intestine (Fig. 3A) or brain (Fig. 3G) of fish fed any of the experimental diets. In contrast, the steady-state level of *fabp7b *mRNAs was higher in intestine of fish fed LND compared to *fabp7b *mRNAs in the intestine of fish fed LD and LFD, but not different in intestine of fish fed HD (Fig. 3B). Also, the steady-state level of *fabp7b *mRNAs was elevated (10-fold) in the brain of fish fed LD compared to the level of *fabp7b *transcripts in brain of fish fed LFD and LND, but was not different in brain of fish fed HD (Fig. 3H).

The steady-state level of *fabp7a *mRNAs was 4-fold higher in the liver of fish fed LFD compared to the transcript levels in the liver of fish fed one of the three other diets (Fig. 3C). The steady-state level of *fabp7a *mRNAs was 3-fold higher in muscle of fish fed LND compared to mRNAs levels for this gene in muscle of fish fed LD, but was not different in the muscle of fish fed HD and LFD (Fig. 3E). No difference was observed in the steady-state level of *fabp7b *mRNAs in liver of fish fed any of the four experimental diets (Fig. 3D). Finally, *fabp7b *mRNAs were not quantifiable by RT-qPCR in muscle (Fig. 3F).

### Effect of diet on steady-state level of *fabp11a* and *fabp11b *mRNAs in different tissues

**Figure 4:**
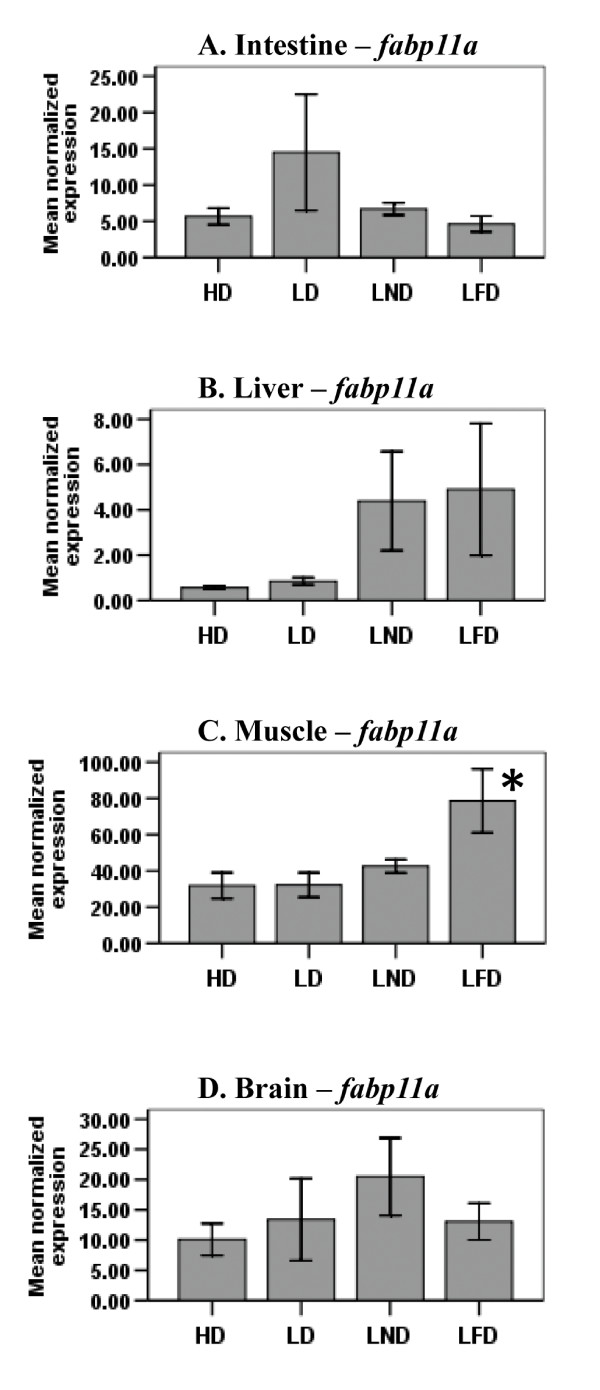
**The steady-state level of *fabp11a *mRNA in intestine (A), liver (B), muscle (C) and brain (D) of zebrafish fed diets differing in FA content**. The level of *fabp11a *mRNA in intestine, liver, muscle, and brain of fish fed either diet HD, LD, LND, or LFD was determined by RT-qPCR using gene-specific primers. The steady-state level of *fabp *transcripts was normalized to the steady-state level of *ef1α *transcripts in the same samples. Data are presented as the mean ratio ± S.E.M. (x 10^-3^). Significant differences in the relative steady-state level of *fabp *mRNA transcripts between fish (n = 6) fed different diets are indicated by an asterisk.

No difference was observed in the steady-state level of *fabp11a *mRNAs in the intestine, liver and brain of fish fed any of the four diets (Fig. 4A, 4B, 4D). The steady-state level of *fabp11a *mRNAs was, however, 2-fold higher in muscle of fish fed LFD compared to *fabp11a *transcript levels in muscle of fish fed the other three diets (Fig. 4C). The level of *fabp11b *mRNAs was not quantifiable by RT-qPCR in liver, muscle and intestine of zebrafish owing to their low abundance. The steady-state level of *fabp11b *mRNAs weas not changed in the brain of zebrafish fed any of the experimental diets (data not shown).

### Modulation of the steady-state level of *fabp *mRNAs is due to up-regulation of transcriptional initiation

Heterogeneous RNA (hnRNA) for a given gene is rapidly processed to a mature mRNA transcript by removal of intervening sequences and the addition of a 5' cap and a poly(A) tail [37]. As such, the level of hnRNA for a given gene indirectly correlates with the rate of initiation of transcription for that gene. To test whether the observed increase in the steady-state level of *fabp *mRNAs by dietary FAs was due to increased transcriptional initiation, the steady-state level of hnRNA for a specific *fabp *gene was assayed by RT-qPCR in different tissues of zebrafish fed the four experimental diets.

**Figure 5:**
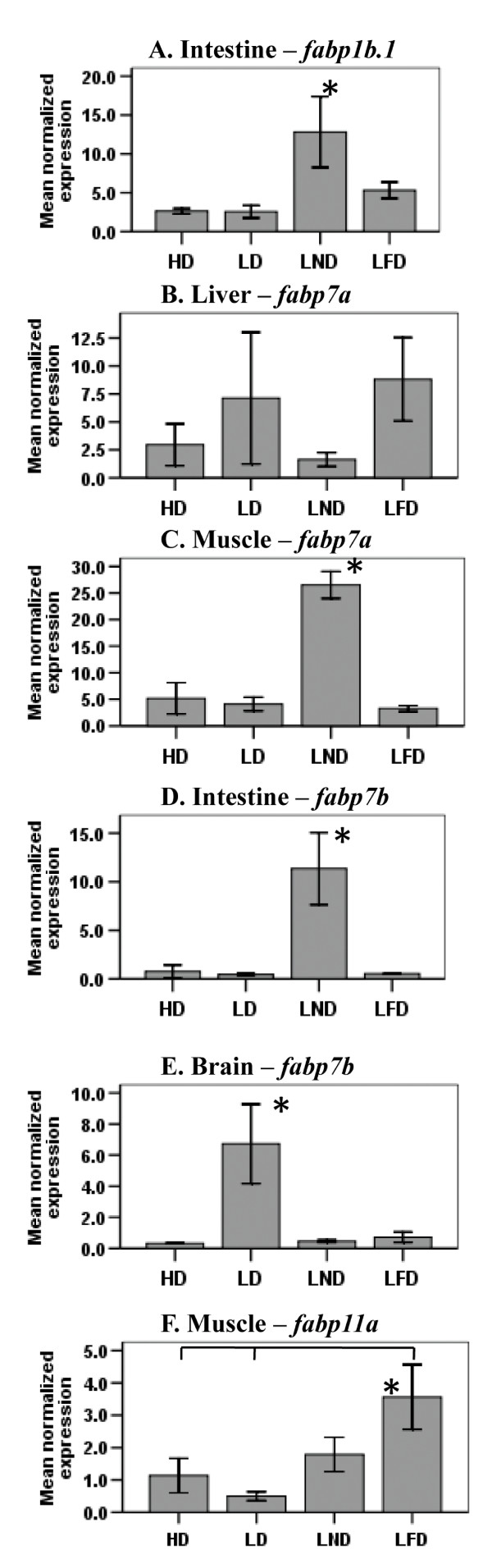
**The steady-state level of hnRNA for *fabp1b.1 *in intestine (A), *fabp7a *in liver (B), *fabp7a *in muscle (C), *fabp7b *in intestine (D), *fabp7b *in brain (E), and *fabp11a *in muscle (F) of zebrafish fed diets differing in FA content**. The levels of hnRNA for *fabp *genes in intestine, liver, muscle and brain of fish fed either diet HD, LD, LND or LFD was determined by RT-qPCR. The steady-state level of *fabp *hnRNA was normalized to the steady-state level of *ef1α *hnRNA transcripts in the same sample. Data are presented as the mean ratio ± S.E.M. (x 10^-3^). Significant differences in the relative steady-state level of *fabp *hnRNA transcripts between fish (n = 5) fed different diets are indicated by an asterisk.

In the intestine of fish fed LND, the level of *fabp1b.1 *hnRNA was higher than in the intestine of fish fed one of the other three diets (Fig. 5A). The steady-state level of *fabp7a *hnRNA was unchanged in the liver of fish fed any of the four experimental diets (Fig. 5B). The level of *fabp7a *hnRNA was higher in muscle of fish fed LND compared to *fabp7a *hnRNA in muscle of fish fed one of the other three diets (Fig. 5C). *fabp7b *hnRNA was elevated in the intestine of fish fed LND compared to *fabp7b *hnRNA in the intestine of fish fed one of the other three diets (Fig. 5D). *fabp7b *hnRNA was 6-fold higher in the brain of fish fed LD compared to fish fed the rest of the treatment diets (Fig. 5E), while the steady-state level of *fabp11a *hnRNA was 2-fold higher in muscle of fish fed LFD compared to hnRNA transcript levels in muscle of fish fed HD and LD, but was unchanged in muscle of fish fed LND (Fig. 5F).

## Discussion

### Effect of diet on FA profiles in tissues of zebrafish

The FA composition in tissues of fish is mediated by various metabolic activities, such as fatty acid desaturation, elongation [38], and β-oxidation [39]. The composition of FAs in tissues is also influenced by dietary lipids [40-42]. In the current study, the FA composition of the four diets fed zebrafish affected the FA profiles of intestine, liver, muscle, and brain (Fig. 1 and additional file 1: Tables S1-S4). Our results are consistent with other studies in which dietary FAs modified the FA profile in zebrafish and other fishes [43-48].

### Dietary FAs modulate the steady-state level of *fabp *mRNAs

Several studies reported the induction of some FABP genes in mammals by FAs and molecular mechanisms for this induction have been proposed [36,49-52]. For example, some research groups [52-54] have suggested that FABPs transport long-chain FAs to the nucleus from the cytoplasm. Once inside the nucleus, FABPs interact with and transfer their long-chain FA ligands to nuclear receptors, such as PPARα and PPARγ [55-57]. Dietary long-chain FAs are known to activate these nuclear receptors [58-62]. Once activated, these nuclear receptors form heterodimers with retinoic acid receptors (RAR) or retinoid X receptors (RXR) (*e.g*., PPAR-RXR or PPAR-RAR), which in turn bind to response elements in *FABP *genes and, thereby, stimulate initiation of transcription [63]. In part, our results showing the increased transcription of *fabp *genes by dietary FAs are consistent with this mechanism of induction (Figs. 2, 3, 4 and 5). However, none of the *fabp *genes were up-regulated in any of the tissues assayed in zebrafish fed HD (Table 2). Indeed, the steady-state level of *fabp1a*, *fabp1b.1 *and *fabp11a *mRNAs was lowest in all of the tissues assayed in fish fed HD (Figs. 2 and 4). Also, the steady-state level of *Fabp7 *mRNAs in rat brain [64], *Fabp5 *mRNAs in mouse liver [65], and the steady-state level of *fabp3 *and *fabp10 *mRNAs in the muscle of Atlantic salmon [47] were not elevated in animals fed diets rich in fish oil. Similarly, Liu *et al*. [66] showed that PC12 cells from rats exposed to eicosapentaenoic acid and docosahexaenoic acid, both abundant in the HD diet fed zebrafish, did not change the level of *Fabp5 *transcripts. Based on the results reported here and previous studies [47,64-66], we propose that, besides up-regulation of *fabp *genes by FAs [67], there may be other mechanisms of transcriptional regulation of the *fabp *genes, such as repression of transcriptional initiation. Berger *et al*. [65] suggested that the observed down-regulation of *Fabp5 *mRNA levels in the liver of mice fed a fish oil diet was mediated via transforming growth factor, beta 1 (TGFβ1). Although a few reports [67-69] emphasize the role of TGFβ1 in the transcriptional regulation of *Fabp *genes, the exact mechanism remains unknown.

**Table 2:** Induction of the steady-state level of mRNA and hnRNA coded by *fabp *genes in tissues of zebrafish fed one of the four diets differing in FA content.

**Diets**	**Intestine**	**Liver**	**Muscle**	**Brain**
HD				
LD				*fabp7b*
LND	*fabp1b.1, fabp7b*		*fabp7a*	
LFD		*fabp7a*	*fabp11a*	

### Differential modulation of *fabp1b.1*, but not *fabp1a *and *fabp1b.2 *transcription by dietary FAs

FAs are known to induce the expression of the *Fabp1 *gene in the mammalian intestine [25,36,49,70,71]. Feeding of high-fat diets rich in vegetable oils resulted in a 30-40% increase in the cytosolic content of FABP1 in the rat intestine [36,49]. Rats weaned to a high-fat diet showed higher levels of *Fabp1 *mRNAs in their intestine than those weaned to a low-fat diet [70,71]. Also, feeding rats a diet containing 6% sunflower oil (rich in linoleic acid) resulted in the greater induction of *Fabp1 *in intestine compared to rats fed a diet containing 3% sunflower oil [25].

Since FAs, such as oleic acid, linoleic acid and linolenic acid, induce the expression of the mammalian *FABP1 *gene [25,26,70,71], we anticipated that the steady-state level of mRNAs for all the duplicated copies of zebrafish *fabp1 *(*fabp1a*, *fabp1b.1*, and *fabp1b.2*), might be modulated by the different FAs in one or more of the experimental diets. However, in this study, we only observed up-regulation of the steady-state level of *fabp1b.1 *mRNAs in the intestine of fish fed LND (Fig. 2) at the level of transcriptional initiation (Fig. 5). The steady-state level of *fabp1a *(Fig. 2) and *fabp1b.2 *mRNAs remained unchanged in the different tissues of zebrafish fed experimental diets. We conclude, therefore, that the *fabp1a *and *fabp1b.2 *genes, but not the *fabp1b.1 *gene, no longer respond to dietary FAs owing to loss of *cis*-regulatory elements in their respective promoters. In their study of *cis*-regulatory elements after a WGD event in yeast, Papp *et al*. [72] concluded that the total number of *cis*-regulatory elements remained unchanged over time between duplicated genes. They further suggest that the number of shared *cis*-regulatory elements between duplicated genes decreased. The difference in the mode of gene regulation reported here for the duplicated *fabp1*s may be due to loss of *cis*-regulatory elements in the *fabp1a *and *fabp1b.2 *genes, a mechanism consistent with the process of subfunctionalization.

In contrast, zebrafish *fabp1*s exhibit a divergent pattern in their tissue-specific distribution of transcripts when compared to their mammalian ortholog. Mammalian *Fabp1 *transcripts were abundant in the intestine, liver and were detected at lower levels in kidney, ovary, and lung [12,73]. In adult zebrafish, *fabp1a *transcripts were detected in the intestine [32], whereas *fabp1b.1 *transcripts were abundant in the intestine [32] but detected at lower levels in the liver, heart, gills, ovary and testes [unpublished data]. Zebrafish *fabp1b.2 *transcripts were abundant in the intestine, ovary and skin, and detected at lower levels in the brain, heart and eye [unpublished data]. This divergent tissue-specific distribution of transcripts for the duplicate *fabp1 *genes differs from previously reported examples of subfunctionalization [3], where the combined tissue-specific expression of duplicated zebrafish genes equals the tissue-specific expression of their mammalian ortholog, as described for the duplicated *hoxb1a *and *hoxb1b *[74], *mitfa *and *mitfb *[75,76], and *sox11a and sox11b *[77] genes. Comparative functional analysis of *cis*-regulatory elements of *Fabp1 *genes will be necessary to determine if the duplicated *fabp1 *genes are retained in the zebrafish genome due to either subfunctionalization or neofunctionalization.

### Tissue-specific transcriptional modulation of *fabp7a *and *fabp7b *mRNAs by dietary FAs

In this study, duplicated copies of zebrafish *fabp7, fabp7a *and *fabp7b*, exhibited distinct patterns of up-regulation by dietary FAs in different tissues for steady-state levels of both mRNA and hnRNA (Figs. 3 and 5). The steady-state level of *fabp7a *mRNAs in muscle and liver were elevated in zebrafish fed LND and LFD, whereas the steady-state level of *fabp7b *mRNAs was modulated in brain and intestine of the fish fed LD and LND.

Mammalian *Fabp7 *transcripts are detected in brain, retina, spinal cord and mammary gland [8,12]. Zebrafish *fabp7a *transcripts are detected in brain, spinal cord, retina, testis, liver, intestine, and muscle [18] and zebrafish *fabp7b *transcripts are detected in brain, retina, testis, liver, intestine, skin, and swim bladder [18]. In addition, the steady-state level of zebrafish *fabp7b *transcripts, but not *fabp7a *transcripts, was up-regulated in the brain of zebrafish fed LD (Fig 3).

Since the duplicated copies of the zebrafish *fabp7*, *fabp7a *and *fabp7b*, show a different tissue-specific pattern of expression compared to their mammalian ortholog and exhibit a different mode of gene regulation by dietary FAs from each other, it is possible that either subfunctionalization or neofunctionalization may account for the retention of both duplicated *fabp7 *genes in the zebrafish genome.

### Transcriptional modulation of *fabp11a*, but not *fabp11b*, by dietary FAs

Previously, we reported that the duplicated copies of the zebrafish *fabp11 *gene and the tetrapod *FABP4, FABP5, FABP8 *and *FABP9 *genes are derived from a common ancestral gene [33]. To date, the *fabp11 *gene has only been identified in fishes [33,78]. This study shows that both the steady-state level of *fabp11a *mRNAs (Fig. 4) and hnRNAs (Fig. 5) was only elevated in muscle of fish fed LFD, but not in muscle of fish fed one of the other three diets. Although *fabp11a *and *fabp11b *exhibit different tissue-specific patterns of expression in embryos, larvae and adult zebrafish [29,33] and differential regulation by dietary FAs, we are unable at this time to resolve whether these duplicated genes were retained in the genome by either subfunctionalization or neofunctionalization as no ortholog of this gene has been identified thus far in other species, such as birds or mammals.

## Conclusion

In this study, we show that dietary FAs change the FA profile in intestine, liver, muscle and brain of zebrafish. The tissue-specific changes in FA content modulated the steady-state level of mRNAs for only one sister duplicate of three pairs of duplicated genes of the *fabp *multigene family in zebrafish (Table 2). Furthermore, changes in *fabp *hnRNA directly correlated with changes in the steady-state levels of *fabp *mRNAs, suggesting that the affects of FAs on these *fabp *genes occurred at the site of transcriptional initiation (Table 2). These findings indicate that the retention of duplicated *fabp *genes in the zebrafish genome is most likely the result of either subfunctionalization or neofunctionalization. To distinguish between these processes as outlined in the DDC model [3] will require functional analysis of the *fabp *promoters to identify *cis-*elements responsible for transcriptional induction by FAs.

## Methods

### Diets and fish husbandry

Four isoproteic (41% crude protein) diets differing in FA composition and lipid content were formulated (Table 1). In addition to National Research Council recommendations on the nutritional requirements of warm-water fishes [79], we were guided by the results of previous dietary studies in zebrafish [43-45,80,81] in the formulation of the diets used in this study. All dry ingredients were mixed in a Hobart mixer. Butyl hydroxy anisole was dissolved in the lipid source and choline chloride was dissolved in distilled water. Both of these solutions were then added to dry ingredients to make a wet dough. The wet dough was scrubbed through an 800 μm screen and the resulting wet particles were freeze-dried and stored at -20°C. The particle size was 600-800 μm in diameter, a size interval for feeding appropriate to the age and weight of the fish used in this study. Mean weight of fish at the start of the feeding trial was 0.26 g. Highly unsaturated FA-rich diet (HD), linoleic acid-rich diet (LD), linolenic acid-rich diet (LND) contained 12% lipid, whereas the low fat diet (LFD) was composed of 4% lipid (Table 1). The FA composition of the four diets is shown in Table 3. The concentration of linoleic acid (18:2 n-6) was highest in LD (44.82%), while the concentration of linolenic acid (18:3 n-3) was highest in LND (33.53%). Among the four diets, the concentration of eicosapentaenoic acid (20:5 n-3) and docosahexaenoic acid (22:6 n-3) was highest in HD (6.14% and 20.94%, respectively).

**Table 3:** Major fatty acids in the experimental diets^1^

**Fatty acid**	**HD^2^**	**LD^2^**	**LND^2^**	**LFD^2^**
12:0	7.73	7.14	6.24	0.80
14:0	3.58	3.19	2.84	1.05
16:0	7.08	6.94	6.65	8.44
18:0	3.36	4.15	3.94	4.15
Total Saturates	23.43	24.80	21.57	16.15
18:1 n-9	11.61	18.12	17.19	17.70
Total Monoenes	16.39	19.03	18.82	20.53
18:2 n-6	17.82	44.82	23.81	39.93
18:3 n-3	8.66	9.44	33.53	20.38
20:5 n-3	6.14	0.04	0.03	0.11
22:6 n-3	20.94	0.14	0.30	0.13
Total PUFA^2^	60.14	56.12	59.68	63.32

^1^Data expressed as area percentage of fatty acid methyl esters.

^2 ^HD - highly unsaturated FA-rich diet; LD - linoleic acid-rich diet; LND - linolenic acid-rich diet; LFD - low fat diet; PUFA - polyunsaturated FAs.

One hundred and eighty wild-type inbred zebrafish siblings (mean wt. 0.26 g), bred from 2 males and 2 females (obtained from a local aquarium supply store) to control for genetic variance, were randomly assigned to 12 aquaria (35 l) with 15 fish per tank at approximately 20 weeks of age. Each dietary treatment had three tank replicates to control for environmental variance. Fishes were acclimatized for 15 days and fed Tetramin^® ^(Tetrawerke, Melle, Germany) twice a day to satiation. The Tetramin diet was discontinued and then fish were fed one of the four diets twice a day at 0900 h and 1900 h to satiation for 10 weeks. A constant water temperature (28°C), a light/dark cycle (14/10 h) and other water parameters were maintained according to Westerfield [82]. Feeding was stopped 24 h prior to sampling of tissue. Fish were individually euthanized by immersion in 0.2% MS-222, weighed and dissected to collect intestine, liver, muscle and brain tissues for RNA and FA analysis. Animal husbandry and protocols for this experiment were reviewed by the Animal Care Committee of Dalhousie University in accordance with the Canadian Council on Animal Care.

### RNA isolation, cDNA synthesis and RT-qPCR

Total RNA was isolated from intestine, liver, muscle and brain of zebrafish using the standard TRIzol method (Invitrogen, Carlsbad, California, USA). The quality and quantity of extracted RNA was assessed by agarose gel-electrophoresis and spectrophotometry at 260 nm, respectively. Messenger RNA in 2 μg of total RNA was converted to cDNA using an oligo(dT) primer according to the manufacturer's instructions for the Omniscript RT kit (Qiagen, Mississauga, Ontario, Canada). cDNA was synthesized from heterogeneous nuclear RNA (hnRNA) using random hexamers. Primer pairs for quantification of mRNA and hnRNA encoded by different *fabp *genes and the annealing temperature for primer pairs for each *fabp *gene are shown in Table 4. For assay of gene-specific hnRNA, one primer was based on an intronic sequence, while the other was based on an exonic sequence (see Table 4 for details). Primers for the amplification of elongation factor 1 alpha (*ef1α*) mRNA by RT-qPCR are based on a previous study [83].

**Table 4:** Primers used for the quantification of *fabp *mRNA and hnRNA

**Gene**	**Forward primer**	**Reverse primer**		
	**mRNA quantification**		**AT^1^**	**Entrez gene ID**
*fabp1a*	TAAGCTGACAGCGTTTGTGAAGGG	AGATGCGTCTGCTGATCCTCTTGT	60.0	791610
*fabp1b.1*	AAGCTGAAGGTGGTGCTGAACA	CACGTTTGCTGATGCGCTTGTA	58.0	554095
*fabp1b.2*	TGCCGTTCTCTGGGAAGTTTGAGT	TGACTTTGTCTCCGCTCAGCATCT	67.5	
*fabp7a*	TGTGCCACTTGGAAACTGGTTGAC	AACATTGCCTACTTGCCTGGTAGG	56.5	58128
*fabp7b*	AAACCACTGCTGATGACCGACACT	AGTGGTCTCTTTCCCATCCCACTT	56.0	407736
*fabp11a*	TGTGCAGAAACAGACCTGGGA	ACAGCCACCACATCACCCATCTT	56.0	447944
*fabp11b*	GCTGTCACTACATTCAAGACCTG	AGTTTACCATCCGCAAGGCTCA	56.0	553579
*ef1α*	TTGAGAAGAAAATCGGTGGTGCTG	GGAACGGTGTGATTGAGGGAAATTC	59.6	30516
	**hnRNA quantification**			
*fabp1b.1*	GAACTAACGTGTGCTGCTTGTG^2^	CACGTTTGCTGATGCGCTTGTA^3^	60.0	554095
*fabp7a*	CCATCCATCAGATTTCTATGTGGG^2^	CATTATGCCTTCTCGTATGTGCG^3^	58.0	58128
*fabp7b*	TTGGAAATGTGACCAAACCGACGC^3^	TCGTCTCGAAAGGGAATGCAGTGT^2^	59.0	407736
*fabp11a*	CCAAGCCGTTTTTGATGATGTGAG^2^	GCTATTAATTTCCCATCCGACACC^3^	61.0	447944
*ef1α*	AGCCTTGCATTCGTGCTGAAGT^2^	GGAACGGTGTGATTGAGGGAAATTC^3^	59.0	30516

^1 ^AT, annealing temperature

Primer based on intronic^2 ^and exonic^3 ^sequences for gene-specific amplification.

The target sequence for each gene was quantified to generate a standard curve of known copy number. Amplification of cDNA samples and DNA standards was carried out using the SYBRGreen Quantitect PCR Kit (Qiagen, Mississauga, Ontario, Canada) following the manufacturer's instructions. For thermal cycling and fluorescence detection, a Rotor-Gene 3000 system (Corbett Research, Sydney, Australia) was used. PCR conditions were as follows: initial denaturation for 15 min at 95°C followed by 40 cycles of 15 s denaturation at 94°C, 20 s annealing of primers at different temperatures depending on the primer pairs (see Table 4), and 30 s of elongation at 72°C. Following the PCR cycles, the melting temperature of the PCR product was determined to assess its purity. Fluorescence was measured following each cycle. The copy numbers of mRNA and hnRNA for each *fabp *gene were determined using the standard curves as explained by Bustin *et al*. [84]. As negative controls, the reverse transcriptase was omitted from cDNA synthesis reactions for each sample and these controls were subjected to RT-qPCR. To determine the relative steady-state level of *fabp *mRNA and hnRNA in each tissue, the absolute copy number of *fabp *mRNA and hnRNA transcripts was divided by the copy number of *ef1α *mRNA and hnRNA transcripts in each sample.

### Lipid extraction, FAME preparation and gas chromatography

A modified Folch procedure [85] described by Budge *et al*. [86] was used to extract neutral lipid fractions from tissues. Briefly, the tissues were homogenized and sonicated for four minutes in 8:4:3 chloroform:methanol:water and the process was repeated four times. Following each extraction, the organic layer was removed, pooled and concentrated under a gentle stream of nitrogen. FA methyl esters (FAMEs) of tissue and dietary lipid were prepared with 7% boron trifluoride in methanol and heating to 100°C for 60 minutes [87]. FAMEs were separated by a gas chromatograph equipped with a flame-ionization detector (Hewlett Packard 6890 GC system, Wilmington, Delaware, USA) on an Omegawax 320 capillary column (30 m × 0.32 mm × 0.25 μm; Supelco, Bellefonte, Pennsylvania, USA). FAMEs were identified by comparison of retention times with those of known standards (Supelco 37 and menhaden oil; Supelco, Bellefonte, Pennsylvania, USA).

### Statistical analysis

Microsoft Excel^® ^2003 and SPSS^® ^14.0 (Chicago, USA) were used for statistical analysis. The relative abundance of mRNA and hnRNA encoded by each *fabp *gene is presented as means ± S.E.M. The significance level was set at *P *< 0.05. The effect of diet on FA composition and the relative abundance of mRNA and hnRNA encoded by each *fabp *gene in different tissues were analyzed by one-way ANOVA. *Post hoc *comparisons were conducted using the Tukey's HSD test.

## List of Abbreviations

FABP: fatty acid-binding protein; iLBP: intracellular lipid-binding protein; WGD: whole genome duplication; FA: fatty acid; HD: highly unsaturated FA-rich diet; LD: linoleic acid-rich diet; LND: linolenic acid-rich diet; LFD: low fat diet; SEM: standard error of means; RT-qPCR: reverse transcription, quantitative polymerase chain reaction.

## Authors' contributions

SK and JMW conceived and designed the research; SK conducted the experimental work and statistical analysis; SPL provided expertise in the design of diets and fatty acid analyses; ED-W assisted in design and interpretation of RT-qPCR analysis; SK and JMW drafted the manuscript with subsequent editorial comments from SPL and ED-W. All authors read and approved the final version of the manuscript.

## Supplementary Material

Additional file 1 **Fatty acid composition in different tissues of zebrafish fed experimental diets**. The data represents the fatty acid composition of the intestine (Table S1), liver (Table S2), muscle (Table S3) and brain (Table S4) of zebrafish fed either diet HD, LD, LND or LFD. Click here for file
