# Supplementary material for: Differential transcriptional modulation of duplicated fatty acid-binding protein genes by dietary fatty acids in zebrafish (Danio rerio): evidence for subfunctionalization or neofunctionalization of duplicated genes
Source: BMC Evol Biol. 2009 Sep 2;9:219. doi: 10.1186/1471-2148-9-219 (PMC2754478; doi:10.1186/1471-2148-9-219)
Supplement: Additional file 1 — Fatty acid composition in different tissues of zebrafish fed experimental diets. The data represents the fatty acid composition of the intestine (Table S1), liver (Table S2), muscle (Table S3) and brain (Table S4) of zebrafish fed either diet HD, LD, LND or LFD. [file 1471-2148-9-219-S1.rtf]

Fatty acid	HD2 	LD2 	LND2 	LFD2 	
12:0	1.95 ± 0.19	1.65 ± 0.13	1.45 ± 0.26	0.61 ± 0.03	
14:0	2.47 ± 0.1	2.28 ± 0.32	1.88 ± 0.23	1.26 ± 0.19	
16:0	22.94 ± 1.19	18.49 ± 1.80	17.20 ± 0.33	27.37 ± 0.58	
18:0	8.43 ± 0.68	10.09 ± 0.53	10.29 ± 0.85	10.63 ± 0.54	
Total saturates	39.52 ± 0.71a	34.70 ± 1.37ab	25.73 ± 1.22b	40.39 ± 0.04a	
16:1 n-7	2.79 ± 0.21	1.15 ± 0.19	1.36 ± 0.12	2.76 ± 0.17	
18:1 n-9	18.35 ± 1.24	21.21 ± 1.72	25.36 ± 0.87	20.84 ± 1.89	
20:1 n-9	0.98 ± 0.13	1.61 ± 0.11	0.97 ± 0.16	1.54 ± 0.15	
Total monoenes	22.79 ± 1.37	24.27 ± 1.98	28.09 ± 0.93	25.78 ± 0.93	
18:2 n-6	15.52 ± 1.30	21.40 ± 1.89	17.63 ± 1.47	18.78 ± 0.72	
18:3 n-3	3.63 ± 0.61	2.74 ± 0.28	9.35 ± 1.4	4.43 ± 0.01	
20:4 n-6	1.08 ± 0.12	3.18 ± 0.23	2.28 ± 0.23	2.25 ± 0.03	
20:5 n-3	3.94 ± 0.23	1.50 ± 0.21	3.99 ± 0.11	0.98 ± 0.12	
22:6 n-3	10.49 ± 0.56	8.32 ± 0.54	11.30 ± 0.42	3.45 ± 0.10	
Total PUFA	37.20 ± 1.23	39.84 ± 0.56	45.94 ± 1.22	33.12 ± 0.86	
                                                                                                                                       Table S1.  Fatty acid composition in intestine of zebrafish fed experimental diets1

1Data expressed as area percentage of fatty acid methyl esters. Results are mean ± S.E. Within a row, means with different superscript letters differ significantly (P < 0.05).
2 HD, highly unsaturated fatty acids rich diet; LD, linoleic acid rich diet; LND, linolenic acid rich diet; LFD, low fat diet; PUFA, polyunsaturated fatty acids. 


Table S2.  Fatty acid composition in liver of zebrafish fed experimental diets1
Fatty acid	HD2 	LD2 	LND2 	LFD2 	
12:0	0.66 ± 0.29	0.53 ± 0.07	1.14 ± 0.08	0.95 ± 0.13	
14:0	1.14 ± 0.08	0.95 ± 0.13	0.66 ± 0.29	0.53 ± 0.07	
16:0	27.06 ± 1.55	30.03 ± 2.37	16.41 ± 0.32	26.75 ± 2.08	
18:0	7.87 ± 0.66	15.02 ± 2.57	16.85 ± 2.86	10.62 ± 1.08	
Total saturates	37.30 ± 0.12	46.69 ± 4.76	39.05 ± 4.39	43.76 ± 5.29	
16:1 n-7	2.85 ± 1.01	2.25 ± 0.42	2.14 ± 0.40	1.66 ± 0.38	
18:1 n-9	21.22 ± 2.06	22.16 ± 0.69	19.60 ± 2.18	29.27 ± 3.02	
20:1 n-9	0.98 ± 0.13b	1.61 ± 0.11a	0.97 ± 0.16b	0.34 ± 0.14c	
Total monoenes	26.45 ± 1.69	24.42 ± 0.86	23.54 ± 1.92	31.27 ± 3.32	
18:2 n-6	12.87 ± 0.51	14.10 ± 2.31	15.98 ± 0.48	11.38 ± 0.40	
18:3 n-3	3.00 ± 0.35b	1.12 ± 0.25c	10.52 ± 0.22a	2.04 ± 0.33b	
20:4 n-6	0.52 ± 0.06b	3.40 ± 0.54a	1.19 ± 0.48ab	1.50 ± 0.39ab	
20:5 n-3	5.50 ± 1.21a	0.67 ± 0.06b	1.02 ± 0.170b	1.08 ± 0.24b	
22:6 n-3	8.28 ± 1.02	4.48 ± 0.85	5.37 ± 1.68	4.33 ± 1.41	
Total PUFA2	35.61 ± 1.81ab	27.72 ± 3.91ab	37.48 ± 2.50a	24.67 ± 2.68b	

1Data expressed as area percentage of fatty acid methyl esters. Results are mean ± S.E. Within a row, means with different superscript letters differ significantly (P < 0.05).
2 HD, highly unsaturated fatty acids rich diet; LD, linoleic acid rich diet; LND, linolenic acid rich diet; LFD, low fat diet; PUFA, polyunsaturated fatty acids. 


Table S3.  Fatty acid composition in muscle of zebrafish fed experimental diets1
Fatty acid	HD2 	LD2 	LND2 	LFD2 	
12:0	1.53 ± 0.30	1.49 ± 0.19	1.53 ± 0.23	0.63 ± 0.11	
14:0	1.86 ± 0.14	1.54 ± 0.13	1.39 ± 0.14	1.01 ± 0.12	
16:0	22.31 ± 0.08a	20.03 ± 0.71ab	17.22 ± 0.44b	18.57 ± 1.16b	
18:0	5.23 ± 0.56	5.92 ± 0.17	6.34 ± 0.74	7.48 ± 0.56	
Total saturates	31.51 ± 1.55	29.99 ± 0.17	29.12 ± 0.48	29.70 ± 1.27	
16:1 n-7	3.03 ± 0.16	1.99 ± 0.38	1.53 ± 0.24	1.77 ± 0.71	
18:1 n-9	24.14 ± 0.37	23.42 ± 0.25	27.15 ± 1.2	29.28 ± 1.72	
20:1 n-9	1.18 ± 0.21	1.15 ± 0.17	1.22 ± 0.22	0.57 ± 0.08	
Total monoenes	29.11 ± 0.33	27.12 ± 0.41	29.35 ± 1.78	32.03 ± 1.56	
18:2 n-6	15.04 ± 0.72b	22.79 ± 0.63a	16.22 ± 0.74b	16.73 ± 0.68b	
18:3 n-3	2.68 ± 0.34b	2.37 ± 0.15b	9.45 ± 0.56a	4.46 ± 0.31b	
20:4 n-6	1.59 ± 0.37b	4.94 ± 0.32a	1.21 ± 0.29b	2.51 ± 0.32ab	
20:5 n-3	4.99 ± 0.31a	1.11 ± 0.2b	2.24 ± 0.36ab	1.15 ± 0.19b	
22:6 n-3	11.45 ± 0.95	7.64 ± 0.86	8.69 ± 0.78	8.76 ± 0.29	
Total PUFA	38.97 ± 1.94	42.57 ± 0.61	41.18 ± 1.26	37.58 ± 1.51	
                                                                                                                                                        
1Data expressed as area percentage of fatty acid methyl esters. Results are mean ± S.E. Within a row, means with different superscript letters differ significantly (P < 0.05).
2 HD, highly unsaturated fatty acids rich diet; LD, linoleic acid rich diet; LND, linolenic acid rich diet; LFD, low fat diet; PUFA, polyunsaturated fatty acids. 


Table S4.  Fatty acid composition in brain of zebrafish fed experimental diets1                                                                                                                                                          
Fatty acid	HD2 	LD2 	LND2 	LFD2 	
12:0	0.46 ± 0.03	0.38 ± 0.11	0.33 ± 0.11	0.34± 0.05	
14:0	1.08 ± 0.12a	0.83 ± 0.13ab	0.59 ± 0.02b	0.76 ± 0.11ab	
16:0	21.64 ± 1.14	20.45 ± 0.33	19.98 ± 0.69	22.48 ± 0.94	
18:0	3.46 ± 0.46ab	3.85 ± 0.46a	1.29 ± 0.67b	3.11 ± 0.22ab	
Total saturates	29.74 ± 1.39a	28.50 ± 0.33ab	24.34 ± 0.96b	29.96 ± 1.57a	
16:1 n-7	0.89 ± 0.06	0.97 ± 0.08	0.91 ± 0.04	1.09 ± 0.09	
18:1 n-7	27.93 ± 1.46	26.03 ± 0.51	26.27 ± 0.55	27.87 ± 0.5	
18:1 n-9	11.11 ± 0.6	11.95 ± 0.7	13.06 ± 0.94	13.19 ± 0.88	
20:1 n-9	0.36 ± 0.03b	0.50 ± 0.01a	0.34 ± 0.01b	0.26 ± 0.03b	
Total monoenes	41.63 ± 1.59	41.93 ± 0.23	43.01 ± 1.28	43.60 ± 1.04	
18:2 n-6	8.11 ± 0.56ab	9.60 ± 1.19a	4.86 ± 0.65b	7.10 ± 1.09ab	
18:3 n-3	1.35 ± 0.11	1.01 ± 0.21	  1.76 ± 0.31	1.39 ± 0.11	
20:4 n-6	1.36 ± 0.08c	3.18 ± 0.21a	1.71 ± 0.09bc	2.10 ± 0.17b	
20:5 n-3	1.78 ± 0.22a	0.50 ± 0.01b	1.40 ± 0.12a	0.49 ± 0.04b	
22:6 n-3	11.28 ± 0.5	11.65 ± 0.56	15.58 ± 0.96	11.17 ± 1.07	
Total PUFA	26.11 ± 1.71	27.19 ± 0.14	29.20 ± 0.84	25.12 ± 1.07	

1Data expressed as area percentage of fatty acid methyl esters. Results are mean ± S.E. Within a row, means with different superscript letters differ significantly (P < 0.05).
2 HD, highly unsaturated fatty acids rich diet; LD, linoleic acid rich diet; LND, linolenic acid rich diet; LFD, low fat diet; PUFA, polyunsaturated fatty acids. 
